# Single-cell RNA-seq analysis of mouse carotid artery under disturbed flow and human carotid plaques identifies key cell populations in atherosclerosis development

**DOI:** 10.1038/s41598-025-07395-7

**Published:** 2025-07-01

**Authors:** Xuyang Liu, Xu Li, Xin Wang, Jiawei Zhao, Chen Liu, Shaochi Wang, Zongping Xia, Yuming Xu

**Affiliations:** 1 Department of Neurology, The First Affiliated Hospital of Zhengzhou University, Zhengzhou, China; 2 Department of Endovascular Surgery, The First Affiliated Hospital of Zhengzhou University, Zhengzhou, China; 3 Henan Key Laboratory of Cerebrovascular Diseases, The First Affiliated Hospital of Zhengzhou University, Zhengzhou, China; 4 Clinical Systems Biology Laboratories, Translational Medicine Center, The First Affiliated Hospital of Zhengzhou University, Zhengzhou, Henan China

**Keywords:** Atherosclerosis, Carotid plaque, Disturbed flow, Single-cell RNA-seq, Senescence, Fibroblast, VEGFA + macrophages, Cardiovascular biology, Cardiovascular diseases, Transcriptomics, Biomarkers

## Abstract

Atherosclerosis tends to occur in regions of disturbed blood flow. This study explored how disturbed flow aggravates atherosclerosis using single-cell RNA-seq (scRNA-seq) datasets from mouse carotid arteries under disturbed flow and human carotid artery plaques. The scRNA-seq datasets were obtained from the GEO (GSE159677, GSE43292, GSE163154, and GSE41571) and SRA (PRJNA722117) databases and were processed using Seurat. Functional enrichment analysis was conducted using Gene Set Enrichment Analysis (GSEA) and Gene Ontology (GO). Single-cell Flux Estimation Analysis (scFEA) was used to analyze cell type-specific changes in metabolism and “transcriptomic noise analysis” to examine senescence. GWAS-significant cardiovascular disease (CVD) risk genes were used to calculate risk gene scores for main cell populations. CellChat and Cytosig were used to analyze cell communication and cytokines. scRNA-seq identified seven cell clusters in mouse arteries: endothelial cells (ECs), vascular smooth muscle cells (VSMCs), fibroblasts, pericytes, macrophages, neutrophils, and T cells. Fibroblasts showed the most pronounced changes, particularly in inflammation and TGF-β signaling pathways. ECs, VSMCs, and fibroblasts had the highest enrichment of CVD risk gene scores, with fibroblasts showing most significant increases in gene risk scores after disturbed flow stimulation. A distinct fibroblast subgroup displayed high enrichment in inflammation and ossification-related pathways. CD36 + positive ECs exhibited significant senescence phenotypes following disturbed flow stimulation. Notable increases in VEGFA^+^ macrophages were discovered in the disturbed flow stimulation group, displaying a pronounced M1 pro-inflammatory phenotype associated with the severity of atherosclerosis and plaque stability. This study systematically elucidated functional changes of cell populations under disturbed flow. CD36^+^ ECs, VEGFA^+^ macrophages, and adventitial fibroblasts play critical roles in atherosclerosis.

**Supplementary Information:**

The online version contains supplementary material available at 10.1038/s41598-025-07395-7.

## Introduction

Atherosclerosis is the leading cause of mortality globally^1^. Its main characteristics include the deposition of low-density lipoprotein cholesterol beneath the endothelium, immune cell infiltration, and the formation of atheromatous plaques^2,3^. Although risk factors for atherosclerosis, such as hypertension, hyperlipidemia, and hyperglycemia, are systemic conditions, atherosclerosis tends to occur in the curved and branching regions of arteries^4^. At these sites, blood flow is characterized by low-magnitude oscillatory shear stress, referred to as disturbed flow^5^. Such abnormal flow patterns can lead to endothelial cell dysfunction and injury, which are the initial steps of atherosclerosis^5^. Mechanical stress is also associated with endothelial cell senescence, which participates in atherosclerosis^6^. Endothelial cell senescence is a process where endothelial cells reach a state of permanent cell cycle arrest due to aging, leading to functional decline and contributing significantly to the development of atherosclerosis. Essentially, senescent endothelial cells promote inflammation and disrupt vascular homeostasis, facilitating the progression of atherosclerotic lesions within the vessel walls^7,8^. Hence, atherosclerosis is known to be associated with endothelial cell senescence, but how blood flow disturbances are involved in the progression of atherosclerosis remains unclear.

Disturbed flow can induce endothelial cell transformation into mesenchymal-like cells (EndMT), such as fibroblasts^9^. Under disturbed flow, endothelial cells can even transform into immune-like cells that participate in atherosclerosis^10^. Moreover, disturbed flow can favor vascular smooth muscle cell (VSMCs) migration and proliferation beneath the intima, causing arterial intima thickening^11^. These VSMCs migrating into the intima layer gradually lose contraction-related markers (Myh11, Acta2, etc.) and transform into a “synthetic” phenotype^12,13^. The use of lineage tracing methods has identified many smooth muscle-derived cells in atherosclerotic plaques that lack contraction-related markers and undergo phenotypic modulation toward fibroblast-like, macrophage-like, osteoblast-like, and endothelial-like cells^13^. Furthermore, extensive infiltration of immune-inflammatory cells into the intima creates an immune-inflammatory microenvironment, aggravating the progression of atherosclerosis^14,15^. However, it is unclear how cellular metabolism and senescence profiles change under disturbed flow and which cell types play the most crucial role in the progression of atherosclerosis.

A common model used to study atherosclerosis is the APOE-/- mouse model fed a high-fat diet in combination with partial carotid ligation, and such mice can develop visible atherosclerotic plaques in the left carotid artery as soon as 3 weeks after modeling^16^. This study performed an integrative analysis of mouse carotid artery datasets under disturbed flow and single-cell transcriptome data of human carotid plaques to identify changes in key cell groups during the development of atherosclerosis.

The results could suggest new insights and therapeutic targets to mitigate or delay the occurrence and progression of atherosclerosis.

## Materials and methods

### Data sources

The carotid partial ligation scRNA-seq dataset was obtained from SRA with the accession code PRJNA722117^17^. The human carotid plaque scRNA-seq dataset was obtained from the Gene Expression Omnibus (GEO) database with the accession code GSE159677^18^. Bulk mRNA arrays were downloaded from GSE43292 ( normal artery versus atherosclerosis plaque)^19^, GSE163154 (intraplaque hemorrhage (IPH) versus non-IPH)^20^, and GSE41571(stable plaques versus ruptured plaques)^21^.

### Single-cell data preprocessing

The scRNA-seq datasets downloaded from the GEO database were reanalyzed using Cellranger (v6.1.2)^22^. Seurat (version 4.02)^23^ was used for downstream analysis, including SCTransform, PCA dimensionality reduction, clustering, and UMAP visualization. Cells with total genes < 300 or > 5000 or with a mitochondrial gene proportion > 10% were identified as low-quality cells and filtered out (Fig. S1A). Doublet cells were identified and removed from the remaining cells by R package DoubletFinder (v2.0.3)^24^. The harmony package^25^ was used to remove the batch effect with the default parameters (Fig. S1C, D). The Seurat package’s function “SCTransform” was used to log-normalize, scale, and find highly variable genes (HGVs)^26^. Afterward, dimensionality reduction was performed on all cells using highly variable genes and the top 20 principal components estimated by an Elbow plot (Fig. S1B). Data clustering was performed using the graph-based clustering method integrated into the Seurat package’s “FindNeighbors” function, employing the top 20 principal components. Subsequently, the “FindClusters” function was applied with a “resolution” parameter value of 0.5. The “RunUMAP” function was employed for visualization with “dims” specified as 1:30.

### Cell-type annotation and differential expression analysis

Differentially expressed genes (DEGs) were identified by applying the Wilcoxon rank-sum test, utilizing the ‘FindAllMarkers’ function in the Seurat package. It involved comparing the differences within each cluster to the combined differences of all other clusters. Genes with *P* < 0.05 were considered as DEGs after Bonferroni correction. These clusters and subclusters were annotated based on the top-ranking DEGs using the canonical marker genes from previous studies^27–29^.

### CVD risk gene enrichment score

Building on the methodology established by^30^, which integrates single-cell transcriptomics with genome-wide association studies (GWAS) to prioritize disease-relevant cell types, cell type-specific highly expressed genes were initially computed using the ‘FindAllMarkers’ function in the Seurat package. Subsequently, the top 200 genes were extracted for each cell type based on decreasing log2FC (fold change) and *P* < 0.05. Then, these genes were intersected with the selected genome-wide association study (GWAS)-significant cardiovascular disease (CVD) risk gene sets^31^ to obtain cell type-specific CVD risk gene sets. For each gene within these sets, a score was assigned: 0 if the expression level was < 1, 1 if it fell between 1 and 2, and 2 if > 2. The scores for all genes within a particular cell type’s set were aggregated to compute the specific CVD risk score for that cell type.

### Detecting metabolic modules and metabolomic changes in each cell type

The scFEA^32^ package was used to infer cell-wise metabolic flux from scRNA-seq data and identify context- and cell types-specific metabolic diversities.

### Transcription noisy analysis

Using the methodology established in prior research^33^, an analysis of transcriptional noise was conducted to measure the extent of cell senescence. For each cell type, at least 10 d-flow (disturbed flow) and 10 n-flow (normal control) cells were assessed. Downsampling was performed on all cells to address variations in total UMI counts and to ensure that each cell had an equivalent total number of UMI counts. The cell numbers were downsampled to ensure an equal representation of both d-flow group and n-flow group cells to address variations in cell-type frequency. Subsequently, genes were categorized into ten equally sized bins according to their mean expression, with the exclusion of the top and bottom bins. Within each group, we selected the genes that exhibited the lowest 10% coefficient of variation. The subsampled raw count data were condensed to include only this specific gene set and underwent square-root transformation. The Euclidean distance between each cell and the respective cell-type mean within each group was computed. This Euclidean distance served as a metric for quantifying transcriptional noise in each cell. In addition, the Euclidean distances were averaged for each mouse, and the transcriptional noise ratio was calculated for the d-flow and n-flow mice. Another approach involved the calculation of Spearman’s correlation coefficients for the down-sampled expression matrices across all genes, considering all possible pairwise cell comparisons within each cell type and group. To maintain consistency with the metric’s sign convention, 1 minus the Spearman correlation coefficient was calculated as the second measure of transcriptional noise. The Wilcoxon’s rank sum test was employed to evaluate the relationship between transcriptional noise and group within each cell type. The resulting p-values were adjusted for multiple testing using the Bonferroni-Hochberg method, utilizing the R function p.adjust().

### Calculation of VSMC phenotype modulation score

The concept of the “VSMC modulation score” was introduced to quantify the phenotypic changes in VSMCs induced by disturbed flow stimulation. This score was computed using the following genes: highly expressed genes associated with phenotype-modulated VSMCs (Lum, Dcn, Fn1, Bgn, Mmp2, Col1a1, and Tnfrsf11b) and genes related to contractile VSMCs (Acta2, Tagln, Myh11, Myl6, and Myl9). The SMC modulation score was defined as SMC Modulation Score = [1 + mean (expression of modulated VSMCs genes)] / [1 + mean (expression of contractile VSMCs genes)].

### PROGENy pathway activity and Cytosig cytokine and chemokine effect analysis

PROGENy^34^ was used to identify pathway activity related to the phenotypes. With this, the activity of 14 classical signal pathways was measured using the top 500 most responsive genes. Considering the pivotal role of cytokines in facilitating intercellular communication during atherosclerosis progression, the investigation was extended into cytokine signaling activity using CytoSig^35^, a cytokine signaling analyzer. CytoSig offers a database of target genes influenced by cytokines and a predictive model of cytokine signaling cascades derived from transcriptomic profiles.

### Cluster-specific transcription regulator analysis

First, Seurat’s “FindAllMarkers” function was used to calculate specific highly expressed marker genes for each macrophage subcluster. Next, the list of DEGs was exported and input into the transcription factor prediction website TRRUST^36^, allowing the prediction of cluster-specific transcriptional regulators.

### Cell-cell communication analysis

The R package “CellChat” (version 1.1.3)^37^ was used across all identified cell types to assess the degree of intercellular communication among different cell types and detect the expression of crucial signaling and receptor ligands. A comparative analysis was conducted to determine variations in communication strength, signaling, and receptor ligands between the Partial carotid ligation (PCL) artery and sham artery.

### Assessing the scores of different phenotypes

Signature genes representing various phenotypes (senescence, senescence-associated secretory phenotype (SASP), cholesterol efflux, lysosome, angiogenesis, phagocytosis, and ossification) were obtained from the Molecular Signatures Database (MSigDB)^38^. Subsequently, the AUCell algorithm was applied with default settings to estimate phenotype-related scores for the selected cell populations using the AUCell package (v1.16.0)^39^.

### Bulk dataset preprocessing and analysis

Raw data were acquired from the GEO database, which provided comprehensive information on the platform, samples, and GEO series (GSE) records. Following data retrieval, the raw gene expression values underwent a log2 transformation. The probes were converted into gene symbols to align with the platform’s normalized data annotation. Probes associated with multiple genes were subsequently excluded from the datasets. The ultimate expression values were determined by averaging the gene expression measurements obtained from multiple probes. Subsequently, the limma package^40^ was used to identify DEGs, while the clusterProfiler package^41^ was employed for conducting comprehensive enrichment analyses including Gene Ontology (GO)and Kyoto Encyclopedia of Genes and Genomes (KEGG) analysis^42–44^.

### Statistical analysis

Student’s t-test, Wilcoxon rank-sum test, and Kruskal-Wallis test were used as appropriate. *P*-values > 0.05 were considered not statistically significant and denoted as “n.s.” *P*-values < 0.05 were represented as follows: *P < 0.05, **P < 0.01, ***P < 0.001, and ****P < 0.0001. For multiple-hypothesis testing in the gene set enrichment analysis (GSEA) analysis, *P*-values were adjusted based on the false discovery rate (FDR).

## Results

### scRNA-seq analysis reveals the seven major cell populations of the mouse carotid artery

**Fig. 1:**
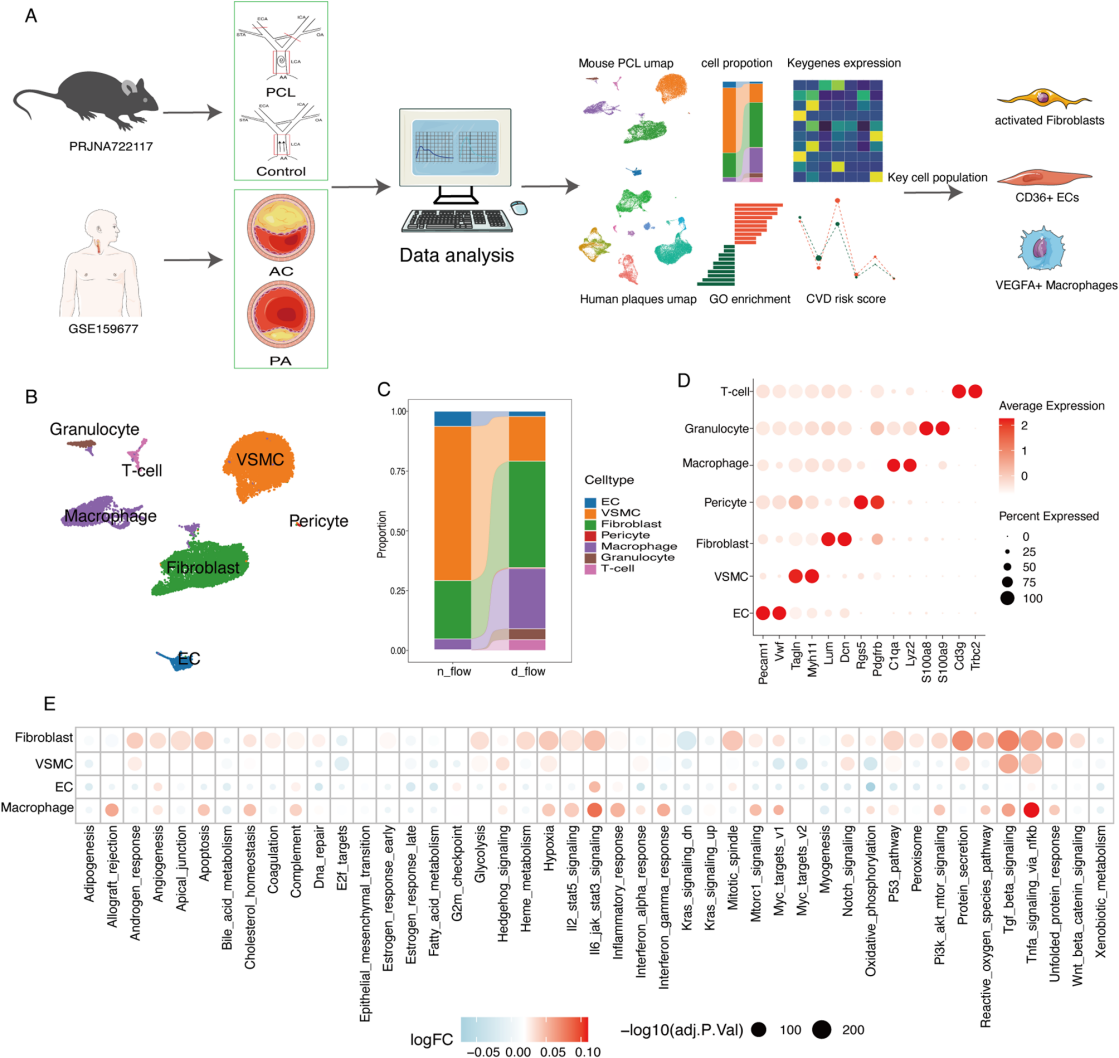
Identification of main cell types in the mouse carotid artery. (**A**) Schematic graph of single-cell RNA sequencing and data analysis pipeline. (**B**) Uniform manifold approximation and projection (UMAP) representation of the landscape of different cell types. (**C**) Stacked barplot of the proportions of cell types in different groups. (**D**) Dotplot showing average expression of known markers in main cell types. (**E**) Dot plot showing differentially enriched pathways in the global cell type between d-flow and n-flow datasets.

Our overall analytical framework is depicted in (Fig. 1A). We initially performed an analysis of the single-cell dataset derived from the mouse carotid artery. After stringent cell filtering to remove low-quality cells and doublets, 8642 cells were retained in the d-flow (PCL) group and 9464 cells in the n-flow (Control) group (Fig. S1A). Following the integration of both datasets, unsupervised clustering was applied to group the cells, and they were visualized using UMAP (Fig. 1B). Cell type annotations were determined using known marker genes, resulting in the identification of seven major cell types (Fig. 1B, D): endothelial cells (Pecam1 and Vwf), VSMCs (Tagln and MYh11), fibroblasts (Lum and Dcn), pericytes (Rgs5 and Pdgfrb), macrophages (C1qa and Lyz2), granulocytes (S100a8 and S100a9), and T cells (Cd3g and Trbc2). In the normal group, the carotid artery was primarily composed of endothelial cells, fibroblasts, and VSMCs, with VSMCs accounting for > 60% of the cell proportion (Fig. 1C), while immune cells, mainly composed of macrophages, made up a small fraction (< 5%). In the disturbed flow group, there was a dramatic increase in the proportion of fibroblasts. In contrast, the proportion of endothelial cells and VSMCs decreased significantly (Fig. 1C). Disturbed flow stimulation induced substantial infiltration of immune inflammatory cells, constituting more than 25% of the total cell count, including monocyte-derived macrophages, neutrophils, and T cells, with monocyte-derived macrophage infiltration being the most prominent. These findings emphasize the crucial role played by immune cells, particularly monocyte-derived macrophages, in the initial stages of atherosclerosis^2^.

To analyze the functional changes in major cell populations following disturbed flow stimulation, gene set enrichment analysis (GSEA) was conducted using the HallMARK GENESET from the Msigdb database (Fig. 1E). Following disturbed flow stimulation, endothelial cells exhibited significant activation of the interleukin (IL)-6-mediated immune inflammatory pathway, confirming that disturbed flow stimulation could induce inflammation in endothelial cells^45^. In addition, the upregulation of the angiogenesis pathway in endothelial cells demonstrated that disturbed flow could activate endothelial cells, promoting their proliferation and migration^46^. VSMCs, on the other hand, showed significant upregulation in pathways such as tumor growth factor (TGF)-β, tumor necrosis factor (TNF)-α, and hypoxia, implying a shift in their phenotype and an enhanced inflammatory state. Macrophages also exhibited clear upregulation in immune-inflammatory pathways, suggesting the important role of macrophage infiltration and the secretion of immune-inflammatory factors in the initiation of atherosclerosis. Surprisingly, fibroblasts, which have not received much attention in previous studies of atherosclerosis, showed significant upregulation in numerous signaling pathways following disturbed flow stimulation, indicating a unique activation state of fibroblasts^47^.

Additionally, we analyzed single-cell sequencing data from human carotid artery plaque samples. We successfully removed batch effects using Harmony (Fig. S2A, B) and classified these cells into 9 major cell types, including endothelial cells, VSMCs, fibromyocytes, macrophages, T cells, NK cells, B cells, plasma cells, and mast cells (Fig. S2C, D).

### Artery stromal cells display a greater enrichment of GWAS-significant risk genes, especially among fibroblasts

**Fig. 2:**
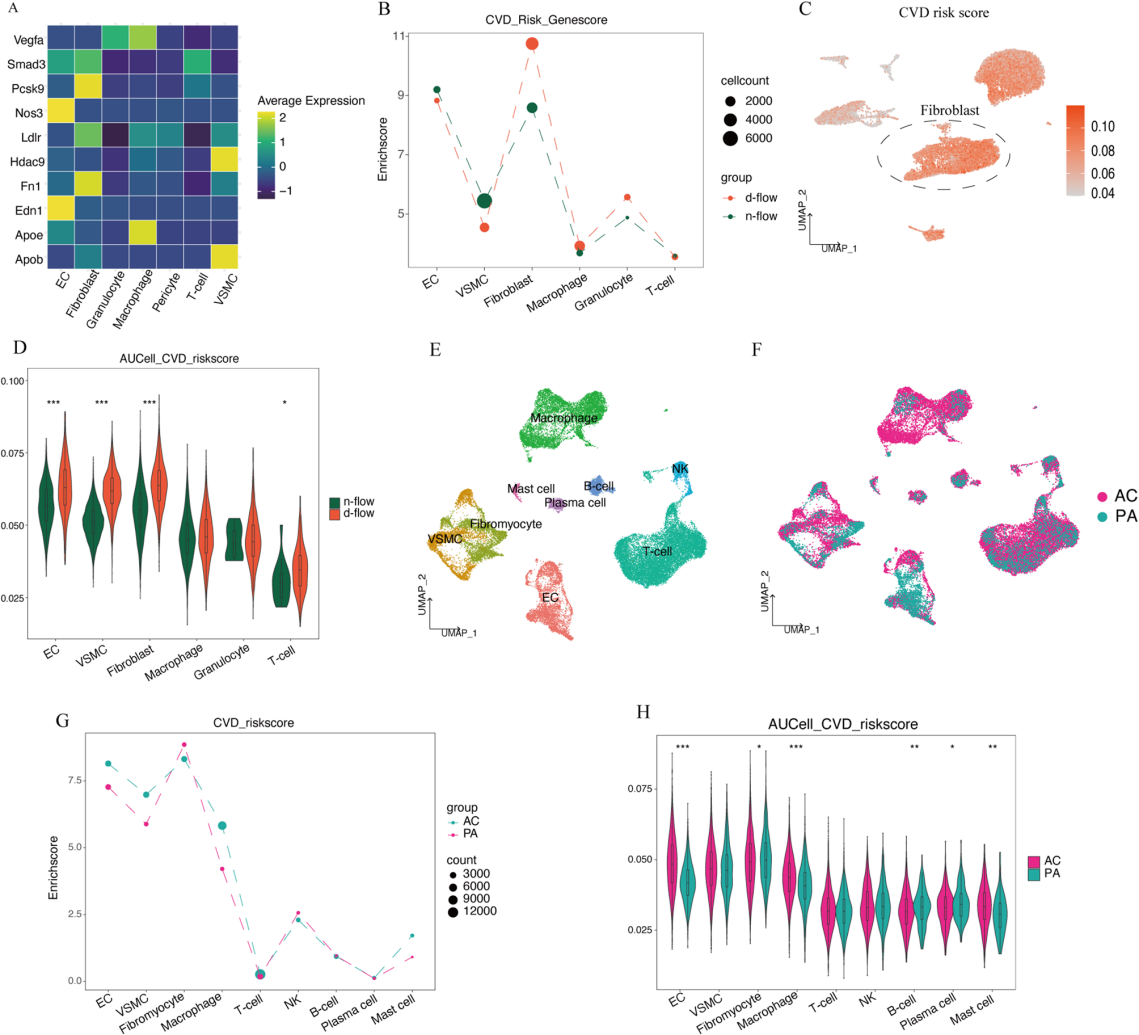
Cardiovascular (CVD) risk genes enrichment difference in main cell types. (**A**) Heatmap of frequently studied CVD risk genes from GWAS studies. (**B**) Dot plot showing the differential CVD risk score in the global cell type between d-flow and n-flow datasets. (**C**) Feature plot of CVD risk score measured by AUCell. (**D**) Vlnplot of CVD risk genes measured by AUCell. (**E**) Major cell populations in the carotid artery plaque are shown by uniform manifold approximation and projection (UMAP). (**F**) UMAP plot of cells from different locations within the carotid plaque. Colors denote different locations. (**G**) Dot plot showing the differential CVD risk score in the global cell type between atherosclerotic core (AC) and adjacent portion (PA) datasets. (**H**) Vlnplot of CVD risk genes measured by AUCell.

In order to provide a comprehensive representation of the enrichment status of GWAS-significant CVD risk genes in mouse carotid arteries subjected to disturbed flow stimulation, cell type-specific CVD risk gene enrichment scores were established. Interestingly, the expression of frequently researched GWAS target genes associated with CVD exhibited cell-specific patterns (Fig. 2A). Edn1 and Nos3 were highly expressed in endothelial cells, Pcsk9 and Fn1 were highly expressed in fibroblasts, Hdac9 and ApoB were highly expressed in VSMCs, and macrophages showed high expression of ApoE and VEGFA.

Based on cell type-specific enrichment scores for CVD risk genes (Fig. 2B), endothelial cells, VSMCs, and fibroblasts had the highest risk gene enrichment scores, especially fibroblasts. After exposure to disturbed flow stimulation, a major high-risk factor for atherosclerosis, the enrichment scores for fibroblasts were significantly higher, further highlighting the unique role of fibroblasts in the occurrence and progression of atherosclerosis. In contrast, immune cells had relatively lower CVD risk gene enrichment scores, suggesting that immune cells are not the primary mode through which genetic factors exert their effects on atherosclerosis. Subsequently, CVD risk genesets were used to generate geneset scores using the AUCell method and then remapped onto UMAP plots. The results are consistent with the above findings (Fig. 2C,D). In addition, after disturbed flow stimulation, there was a significant upregulation of the CVD risk genes enriched in endothelial cells, VSMCs, and fibroblasts (Fig. 2D).

In order to understand the distribution of CVD risk genes within human , the cell types were first grouped and annotated within carotid artery plaques (Fig. 2E,F). In human carotid artery plaques, the major cell types were similar to mouse carotid arteries. Because carotid artery endarterectomy only removes the intima and media of the artery, no fibroblasts derived from the outer layer were found (Fig. 2G). In addition, using the same method, cell type-specific CVD risk gene enrichment scores were constructed for the main cell populations of human plaques. Consistent with previous results, CVD risk gene enrichment scores were the highest in endothelial cells, VSMCs, and fibromyocytes. A higher CVD risk gene enrichment was observed in macrophages in human carotid plaque compared with mouse carotid artery (Fig. 2B, G).

Similarly, the CVD risk genes score for the main cell type calculated by the AUCell method showed consistent results (Fig. 2H). Furthermore, in comparison with the plaque’s marginal region (PA), the atherosclerotic core (AC) of the plaque exhibited a significantly higher enrichment score of CVD risk genes in endothelial cells and fibroblasts, indicating the crucial role of endothelial cells and fibroblasts in the progression of atherosclerosis. Taken together, the results suggest that the artery stromal cells display a greater enrichment of GWAS-significant CVD risk genes, especially among fibroblasts and fibromyocytes, both in mice and humans.

### Fibroblasts show the most prominent metabolic change following disturbed flow stimulation

To illustrate the comprehensive changes in the metabolic state of cell populations following disturbed flow stimulation, scFEA was employed for metabolic functional analysis. scFEA utilizes the key gene expression of a series of metabolic nodes and employs neural network methods to infer the activity of metabolic pathways and the abundance of metabolites.

**Fig. 3:**
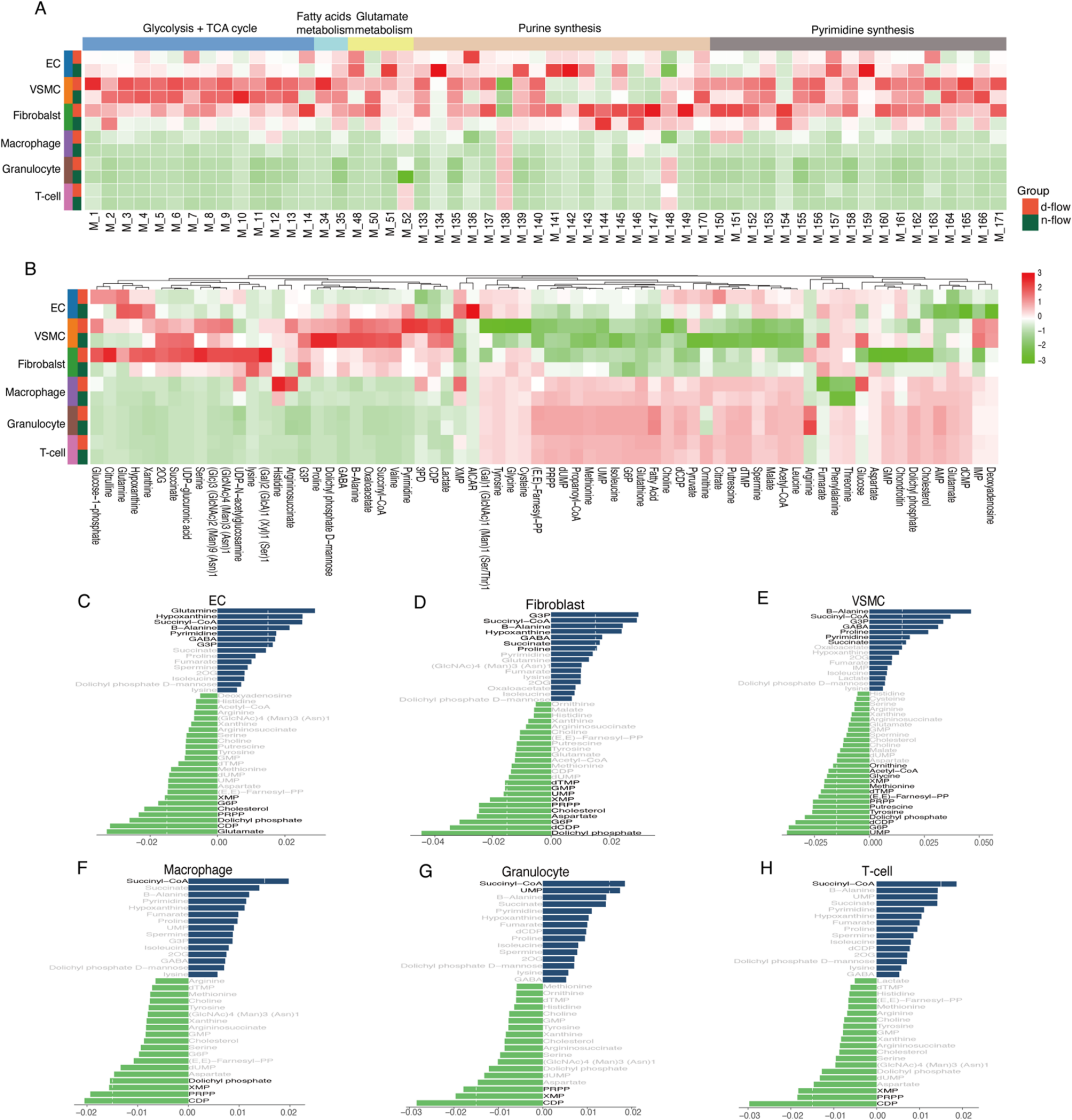
Single-cell metabolic flux mapping of the carotid artery reveals cell type-specific metabolic heterogeneity. (**A**) Heatmap of the predicted flux of metabolic modules. (**B**) Heatmap of the predicted metabolites. (**C–H**) Top accumulated and depleted metabolites in main cell types. The x-axis is the metabolism stress level, where a positive value represents accumulation, and a negative value represents depletion. The dashed line shows the value of accumulated or depleted metabolites equaling 0.015. Values less than this value are represented by gray bars.

Prior research showed that disturbed flow stimulation enhanced glycolytic activity in endothelial cells, while laminar flow had the opposite effect^47,48^. Changes in glycolysis and oxidative phosphorylation in endothelial cells after disturbed flow stimulation were observed, with an increase in the critical glycolytic pathway M4 (3PD-pyruvate) and a general decrease in oxidative phosphorylation-related pathways (M7-M14) (Fig. 3A). They exhibited significant upregulation in numerous metabolic pathways, such as glycolysis, oxidative phosphorylation, and purine and pyrimidine metabolism, which may indicate an active state of cell growth and division, involving substantial DNA and protein replication (Fig. 3A). The results revealed that, overall, different cell types exhibited distinct primary metabolic fluxes (Fig. 3A). Endothelial cells, VSMCs, and fibroblasts generally demonstrated a more active metabolic state. In contrast, immune cells exhibited lower metabolic fluxes, indicating significant differences in metabolism and energy utilization between immune and stromal cells.

The disturbed flow stimulation group also exhibited increased accumulation of pyruvate; surprisingly, the cell type with the most significant metabolic pattern changes induced by disturbed flow was fibroblasts (Fig. 3B). After disturbed flow stimulation, the metabolic modules of VSMCs and fibroblasts became more similar, possibly implying a phenotypic transformation in VSMCs. Furthermore, different cell types also showed notable differences in the accumulation and clearance of metabolites (Fig. 3B).

Endothelial cells, VSMCs, and fibroblasts tended to accumulate more G3P. At the same time, dolichyl phosphate was the most consumed metabolite in these cell types (Fig. 3C–E). In contrast, among immune cells, succinyl-CoA was the most accumulated metabolite, while CDP was the most consumed one (Fig. 3F–H).

### Disturbed flow induces an activation state in fibroblasts

**Fig. 4:**
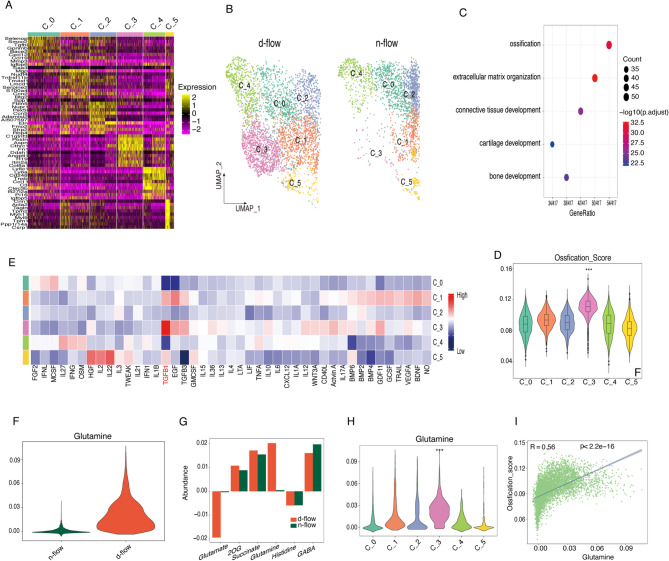
Adventitial fibroblasts exhibit notable functional changes following exposure to disturbed flow, and the C_3 ossification-like fibroblasts display a positive association with glutamate metabolism. ((**A**)) Heatmap depicting the top 10 differentially expressed genes among fibroblast subclusters. (**B**) Uniform manifold approximation and projection (UMAP) plot illustrating the distribution of fibroblast subclusters across different experimental groups. (**C**) GO enrichment analysis specifically targeting the top differential genes within cluster C_3. (**D**) Violin plot presenting the ossification scores of fibroblasts subclusters, offering insights into score distribution. (**E**) A heatmap demonstrating cytokine activity within fibroblast subclusters was computed using the “cytosig” method. (**F**) Vlnplots highlighting Glutamine abundance. (**G**) Barplot illustrating the abundance of metabolites associated with glutamate flux. The Y-axis reflects the level of metabolic stress, with positive values indicating accumulation and negative values indicating depletion. (**H**) Violin plot presenting the abundance of glutamine among fibroblasts subclusters. (**I**) An assessment of the correlation between glutamate abundance and the ossification score.

After performing unsupervised dimensionality reduction and clustering of fibroblasts, six subgroups were identified after disturbed flow stimulation, including a newly emerged subgroup, C_3 (Fig. 4A, B, Fig. S3A). First, differential analysis and subsequent functional enrichment analysis were conducted on fibroblasts from the d-flow and n-flow groups (Fig. S3B). The upregulated signaling pathways primarily converged on inflammation, including TNF, IL-17, and pathways related to inflammation and atherosclerosis, as well as shear stress and atherosclerosis (Fig. S3C). Surprisingly, the downregulated pathways were mainly associated with smooth muscle cell contraction. It suggested that under normal conditions, fibroblasts may also possess a contractile function, potentially playing a role in vascular constriction and relaxation.

To further investigate the functional differences among the fibroblast subgroups, Progeny was used to analyze the pathway activity within each fibroblast subgroup (Fig. S3D). The results indicated that the newly identified C_3 subgroup exhibited the highest activity in the TGF-β and WNT pathways. In addition, top genes in the C_3 subgroup were significantly enriched in ossification-related biological processes (Fig. 4C). Using gene set scoring with ossification-related gene sets, significant ossification-related characteristics were confirmed in the C_3 subgroup (Fig. 4D). Previous research has indicated a clear association between vascular calcification and vascular stiffening^49,50^, suggesting that the C_3 subgroup of fibroblasts may be involved in the atherosclerotic process by inducing vascular calcification.

Overall vascular expression of TGF-β1 was significantly upregulated following disturbed flow stimulation (Fig. S3E), with a surprising observation that TGF-β1 expression was primarily located in macrophages (Fig. S3F). To explore how fibroblasts primarily located in the outer layer are influenced by disturbed flow stimulation and subsequently activate the TGF-B and WNT pathways, Cytosig was used to investigate the role of specific cytokines and chemokines in the emergence of fibroblast subtypes. Consistent with the Progeny findings of TGF-β pathway activation, Cytosig revealed that the C_3 subgroup was significantly stimulated by the transcription factor TGFB1 (Fig. 4E). This phenomenon suggests that the TGF-β1 coming from macrophages play a crucial role in the phenotypic changes of fibroblasts and, consequently, in the progression of arterial stiffness and atherosclerosis.

Based on the previous scFEA analysis, we further explored whether changes in fibroblasts metabolites associated with the emergence of the C_3 subgroup. Surprisingly, compared with the n-flow group, the d-flow group exhibited a significant increase in glutamate consumption and a much higher accumulation of glutamine (Fig. 4F, G, Fig. S3G). The up-regulation in the metabolic flux from glutamate to glutamine (M_48) indicated an increase in glutamine synthesis.

The expression of glutamine in fibroblast subgroups was further analyzed, and the most pronounced accumulation of glutamine was seen in the C_3 subgroup (Fig. 4H). Consistently, the results of correlation analysis indicated a clear positive correlation between glutamine accumulation and the presence of the C_3 subgroup (Fig. 4I). Hence, the emergence of the C_3 subgroup, which is associated with calcification, was observed after being exposed to disturbed flow stimulation. TGF-β1 may stimulate this cell subgroup, and macrophages are the most likely source of this cytokine. In addition, the accumulation of glutamine may also play a crucial role in this process.

### Disturbed flow stimulation induces the aging of endothelial cells

**Fig. 5:**
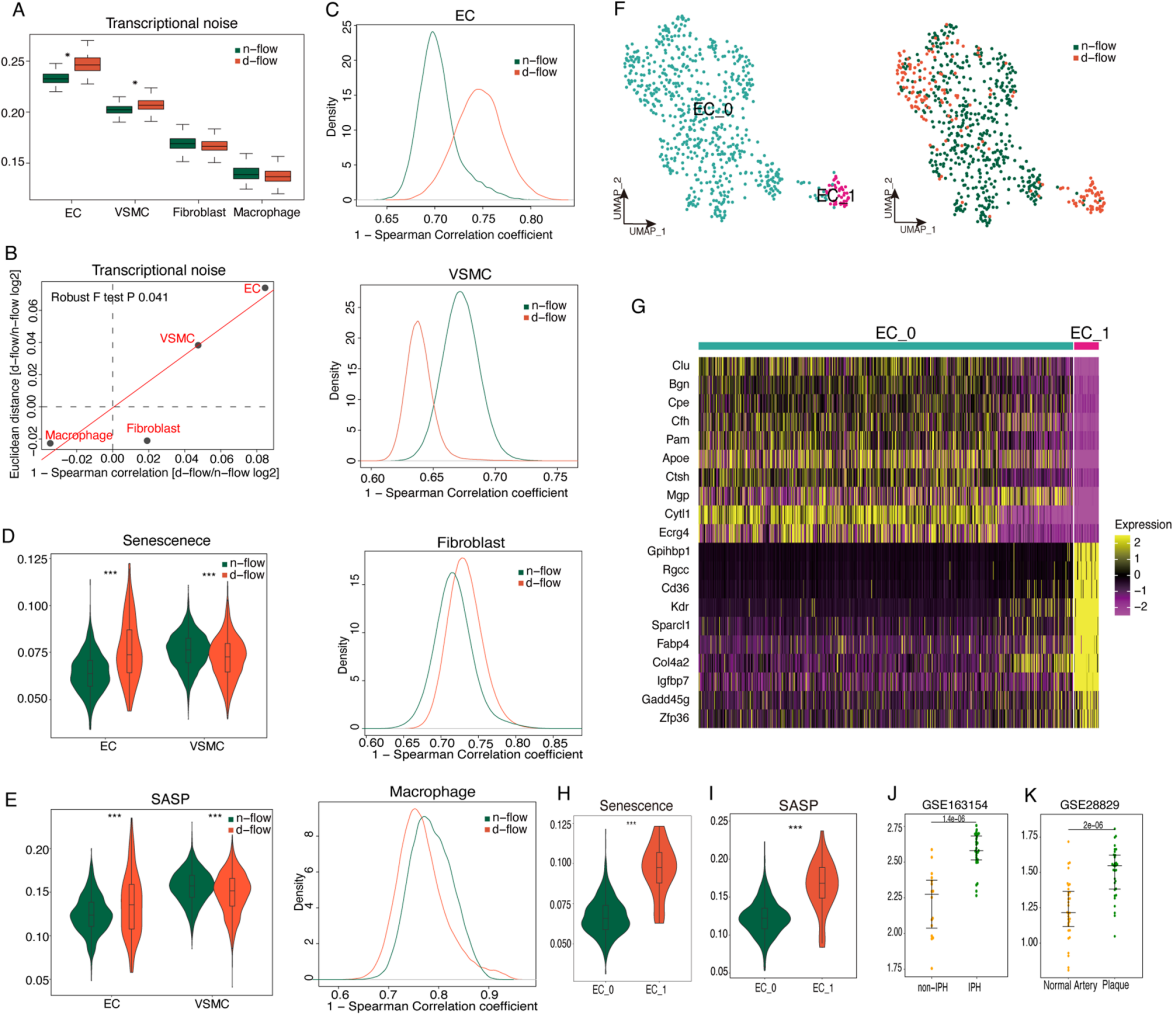
Endothelial cells show the most prominent increase of transcriptional noise and senescence phenotype following disturbed flow, and endothelial cells show distinct internal heterogeneity in senescence. (**A**) Boxplot shows transcriptional noise by different stimulation types and cell types. (**B**) Scatterplot depicts the log2 ratio of transcriptional noise between d-flow and n-flow samples as calculated using the 1-Spearman correlation and the Euclidean distance between cells on the X and Y axes, respectively. (**C**) The distribution of 1-Spearman correlation coefficients between d-flow and n-flow group cells is shown for endothelial cells. The distribution of 1-Spearman correlation coefficients between d-flow and n-flow group cells is shown for smooth muscle cells. The distribution of 1-Spearman correlation coefficients between d-flow and n-flow group cells is shown for macrophages. The distribution of 1-Spearman correlation coefficients between d-flow and n-flow group cells is shown for T cells. (**F**) Uniform manifold approximation and projection (UMAP) plot of endothelial cell subclusters and endothelial cell subclusters grouped by d-flow and n-flow. (**G**) Heatmap of top 10 differential genes in endothelial cell subclusters. (**H**) Vlnplot of Senescence Score in Ec_0 and Ec_1. (**I**) Vlnplot of SASP score in Ec_0 and Ec_1. (**J**) CD36 + Ec signature score of paired early and advanced lesions, intraplaque hemorrhage (IPH), and non-IPH plaques. (**K**) CD36 + Ec signature score of paired early and advanced lesions, IPH (intraplaque hemorrhage), and non-IPH plaques.

Senescence is a significant driving factor in the occurrence and development of atherosclerosis, and it remains unclear whether disturbed flow stimulation can induce senescence and which cell types experience the most pronounced senescence. As senescence progresses, cells exhibit significant transcriptional instability, meaning there is an increasing probability of unstable mRNA^51^. Here, the transcriptional noise was characterized within cell populations by calculating the Euclidean distance within the same cell population. We observed that endothelial cells exhibited the most pronounced senescence phenotype, followed by VSMCs, while fibroblasts and macrophages did not show significant changes (Fig. 5A). In order to further support this discovery, transcriptional noise was measured using an alternative approach by analyzing Spearman’s correlations between cells (Fig. 5B). The analysis confirmed that transcriptional noise was increased most prominently in endothelial cells and VSMCs after disturbed flow stimulation (Fig. 5C). Senescence-related gene sets were selected from Msigdb and used AUCell to make gene set scoring. The results indicated that after disturbed flow stimulation, endothelial cells showed the most significant upregulation in senescence-related gene scores (Fig. 5D) and SASP (Fig. 5E). Therefore, the results suggest that endothelial cell senescence is activated by a disturbed flow.

### CD36^+^ endothelial cells are important contributors to endothelial senescence

To further explore the mechanisms possibly involved in endothelial cell senescence, endothelial cells were isolated and underwent additional dimensionality reduction, clustering, and subgrouping to explore the heterogeneity of senescence within endothelial cells (Fig. 5F,G). Endothelial cells were divided into two cell subgroups: Cytl1 + EC_0 and Cd36 + EC_1 (Fig. 5F,G). CD36^+^ endothelial cell is a novel subgroup that emerged under disturbed flow stimulation, and previous research has indicated that CD36^+^ endothelial cells are primarily found in disturbed flow regions in vivo, such as the aortic arch (Fig. 5F)^51^. Using a senescence gene set scoring approach, it was observed that CD36^+^ endothelial cells exhibited a pronounced aging phenotype (Fig. 5H, I).

The top 100 highly expressed genes in CD36^+^ endothelial cells were used as a gene set to score the bulk data sets of two carotid artery plaques to explore the unique senescent endothelial cells within this group and their relationship with the development of arterial plaques and intraplaque hemorrhage. The results showed that compared with early-stage plaques, advanced-stage plaques exhibited higher expression of this gene set, suggesting a positive correlation between a higher degree of endothelial cell senescence and plaque progression (Fig. 5J). Furthermore, compared with plaques without hemorrhage, the hemorrhagic plaques had higher gene set scores (Fig. 5K). In conclusion, disturbed flow induced a distinct senescence CD36^+^ endothelial cell population, and this senescent cell population was closely associated with the progression of atherosclerosis and plaque hemorrhage.

### Smooth muscle cells undergo prevalent phenotype modulation following disturbed flow stimulation

VSMCs are an important group of cells that make up the blood vessel wall and are closely associated with the occurrence and development of atherosclerosis. Under the influence of risk factors for atherosclerosis, such as a high-fat diet, VSMCs can transition from classic contractile smooth muscle cells to secretory VSMCs (myofibroblasts), cartilage-like smooth muscle cells, and even lipid-engulfing macrophage-like smooth muscle cells. These cells play a crucial role in plaque formation^52^.

**Fig. 6:**
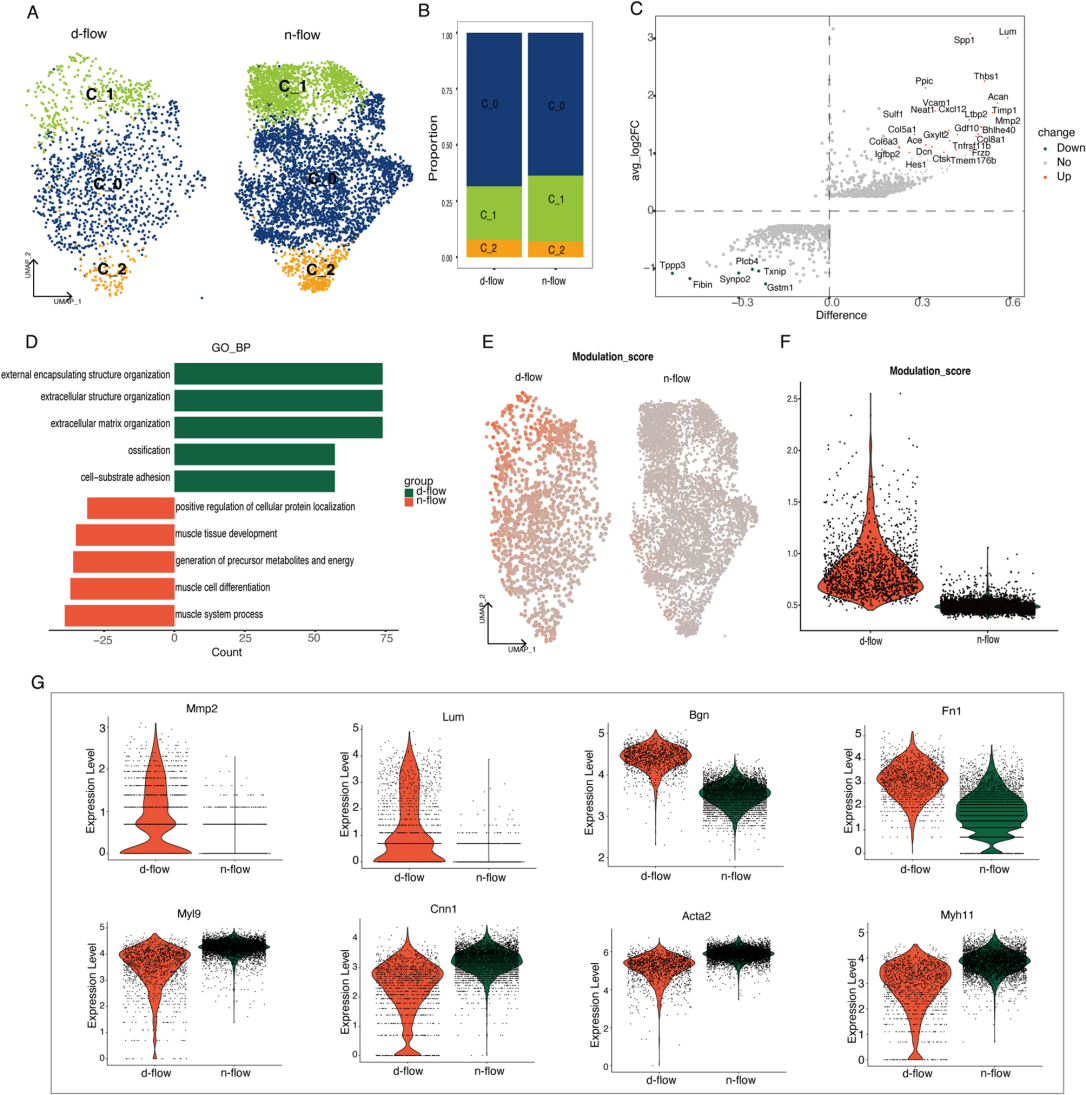
Disturbed flow induced significant phenotype modulation in vascular smooth muscle cells (VSMCs). (**A**) Uniform manifold approximation and projection (UMAP) plot of VSMC subclusters. (**B**) Stacked barplot of VSMC subclusters in d-flow (disturbed flow) and n-flow (normal control) groups. (**C**) Volcano plot revealing differences in gene expression in VSMCs. Differential expression genes with p_val_adj < 0.05 were then selected for analysis. The top differential expression genes are highlighted in the figure. (**D**) Gene Ontology (GO) enrichment of upregulated and down-regulated genes. (**E**) Feature plot showing VSMC modulation score. (**F**) Vlnplot showing VSMC modulation score. (**G**) Vlnplot of classical markers of synthetic (upper panel) and contractile VSMCs (lower panel).

After performing dimensionality reduction and clustering, VSMCs were divided into three subgroups (Fig. 6A). The cells in these three subgroups showed no significant distribution differences between the d-flow and n-flow groups (Fig. 6B). Functional enrichment analysis of these subgroups also did not reveal significant functional differences. As shown in the volcano plot (Fig. 6C), genes related to extracellular matrix mechanisms were significantly upregulated (Lum, MMP2, FN1, and Bgn), while genes related to muscle contraction were downregulated (Myh11, Acta2, Cnn1, and Myl9). The results of the GO enrichment analysis indicate a significant upregulation of biological processes related to the extracellular matrix, such as “extracellular matrix organization,” in the disturbed flow group (Fig. 6D). Conversely, there was a significant downregulation in processes associated with SMC contraction and muscle cell development, such as “muscle tissue development” and “muscle system process.” (Fig. 6D). The “phenotype modulation score” was calculated for VSMCs to explore whether phenotypic modulation in VSMCs is universal or only exist in some cells.

Under disturbed flow stimulation, VSMCs exhibited widespread phenotypic modulation, while cells in the normal control group showed almost no significant phenotypic modulation (Fig. 6E,F). The downregulation of a series of smooth muscle contraction genes and the upregulation of markers for fibroblast-like smooth muscle cells support the above observations (Fig. 6G). However, these SMC subtypes do not form distinct clusters, possibly due to the shorter duration of disturbed flow stimulation compared with atherosclerotic plaques.

### VEGFA^+^ macrophages exhibit a distinct M1-like pro-inflammatory phenotype

**Fig. 7:**
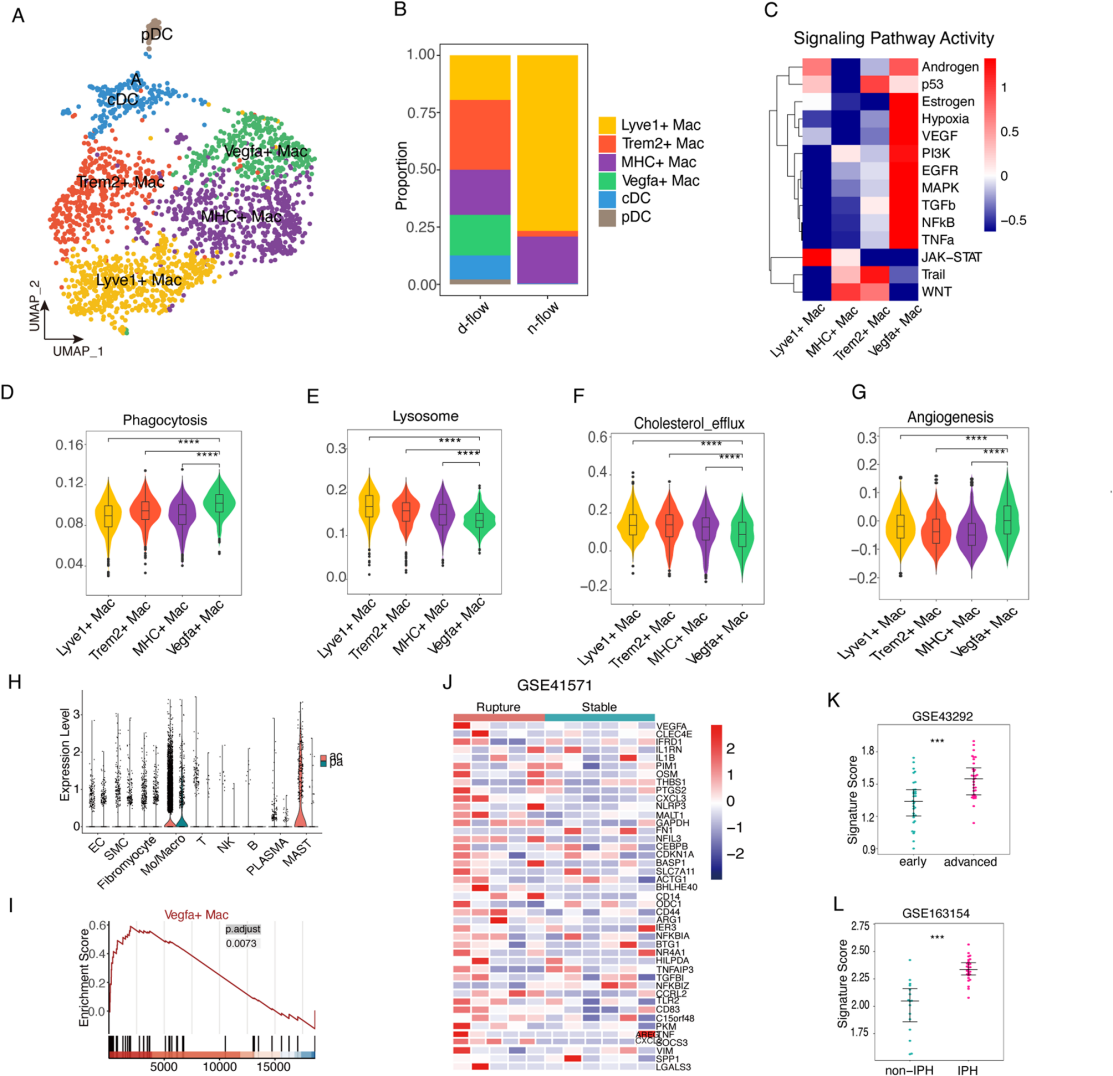
Characteristics of macrophage infiltration induced by disturbed flow and the vascular endothelial growth factor a (VEGFA)^+^ macrophage signature exhibit marked distinctions between rupture and stable plaques. (**A**) Umap plot depicting the main subclusters. (**B**) Stacked barplot illustrating the proportion of main cell types within different groups. (**C**) Heatmap showcasing the activity of signaling pathways, calculated using Progeny. (**D–G**) Vlnplot showcasing four crucial functional scores (Lysosome, Cholesterol efflux, Phagocytosis, and Angiogenesis). (**H**) Gene set enrichment analysis (GSEA) of the VEGFA^+^ macrophage signature. (**I**) Vlnplot displaying VEGFA expression across the primary cell types within human plaques. (**J**) Heatmap revealing the top 50 signature genes associated with VEGFA^+^ macrophages. (**K**) and (**L**) Jitterplots depicting the VEGFA^+^ signature score in paired early and advanced lesions (upper) and between non-intraplaque hemorrhage (IPH) and IPH plaques (lower).

After reclustering and annotation of the macrophages, six distinct cell populations were identified, including two dendritic cell populations (pDc and cDC) and four macrophage populations (VEGFA+, Trem2+, Mhc+, and Lyve1+) (Fig. 7A). In d-flow group, Lyve1 + macrophages were the predominant population within artery, aligning with their functional definition as adventitial-resident macrophages. Notably, Trem2 + macrophages exhibited a significant increase after disturbed flow stimulation (Fig. 7B). Previous research suggested that Trem2 + macrophages may be associated with lipid engulfment and could serve as precursors for foam cells. The elevation of this cell subgroup could indicate disruption of the endothelial barrier function, lipid deposition, and potential lipid phagocytosis induced by disturbed flow. Surprisingly, a specific population of macrophages that highly expressed VEGFA after disturbed flow stimulation was identified (Fig. 7B, Fig. S4B). Transcription factor regulation analysis of the four macrophage subgroups revealed distinct transcriptional regulatory networks for each subgroup (Fig. S4A). Furthermore, using Progeny pathway activity analysis, it was found that VEGFA + macrophages exhibited significantly higher activity in NF-kB, TNF-α, VEGF, and hypoxia-related pathways compared with other cell subgroups, indicating a pronounced pro-inflammatory role for the VEGFA + cell subgroup (Fig. 7C). Traditionally, macrophages are classified into two major functional phenotypes: the pro-inflammatory M1 and the anti-inflammatory M2. In our study, VEGFA⁺ macrophages exhibited significantly higher expression of M1-associated markers such as Tnf-a and Il1b (Fig. S4E), while Lyve1⁺ macrophages showed notable upregulation of key M2 markers, including Cd163 and Mrc1 (Fig. S4F). Consistent with this classification, the glycolysis pathway—commonly linked to the M1 phenotype^50^—was significantly enriched in VEGFA⁺ macrophages (Fig. S4C). In contrast, the oxidative phosphorylation pathway, typically associated with M2 macrophages, was enriched in Lyve1⁺ macrophages (Fig. S4D). These findings suggest that disturbed flow induces a pronounced infiltration of pro-inflammatory, M1-like VEGFA⁺ macrophages. Moreover, the shift in the balance between M1-like VEGFA⁺ and M2-like Lyve1⁺ macrophages may play a critical role in the progression of atherosclerosis.

### The VEGFA^+^ macrophage signature exhibits marked distinctions between rupture and stable plaques

Lipid engulfment is a crucial step in macrophage involvement in atherosclerosis and plaque formation^1^. Through analysis of key pathways related to lipid processing, we discovered that VEGFA^+^ macrophages exhibited the strongest lipid engulfment capacity (Fig. 7D). However, they showed significant weaknesses in two essential lipid processing steps, lysosomal function and lipid efflux, compared with other cells (Fig. 7E, F). This result suggests that VEGFA^+^ macrophages can engulf large amount of lipid but struggle with lipid efflux, leading to the formation of foam cells and the development of necrotic cores.

We further conducted Gene set enrichment analysis (GSEA) using the VEGFA^+^ macrophage TOP 50 genes as geneset to characterize the role of VEGFA^+^ macrophages in atherosclerosis. The results showed higher expression of VEGFA^+^ macrophages signature genes in vulnerable plaques compared to stable plaques (Fig. 7J). Furthermore, the distribution of VEGFA^+^ macrophages was examined in human carotid artery plaques. The results revealed presence of substantial number of VEGFA^+^ macrophages in human carotid artery plaques, especially in core of the plaques (AC) compared with the plaque periphery (PA) (Fig. 7H). Additionally, when correlated with the clinical features of the plaques, the VEGFA^+^ macrophage signature score was higher in the advanced areas of plaques compared with relatively normal arteries (Fig. 7K). Consistently, compared with non-hemorrhagic plaques, hemorrhagic plaques exhibited higher gene set scores (Fig. 7L). This phenomenon may be related to the promotion of vascular angiogenesis by VEGFA^+^ macrophages within the plaques (Fig. 7G), leading to increased neovascularization within the plaque, making it more prone to rupture and bleeding.

### Infiltrating T lymphocytes exhibit unique functional heterogeneity

T cells play key roles in atherosclerosis^53^. In normal artery, the number of T cells was quite low, but after disturbed flow stimulation, there was a significant increase in T cell infiltration (Fig. S5A, C). Those cells were reclassified, annotated, and divided into four main groups: CD4 + T cells, CD8 + T cells, γ_δ T cells, and a subset of NK cells (Fig. S1B, D). Among them, CD4 + T cells exhibited the highest upregulation of inflammation-related signaling pathways, while γ_δ T cells showed upregulation in early hypoxia-related pathways (Fig. S1E). These results suggest that disturbed flow induces T-cell recruitment in the arterial wall, where they can participate in atherosclerosis.

**Cell communication presents an active state under disturbed flow stimulation.**

**Fig. 8:**
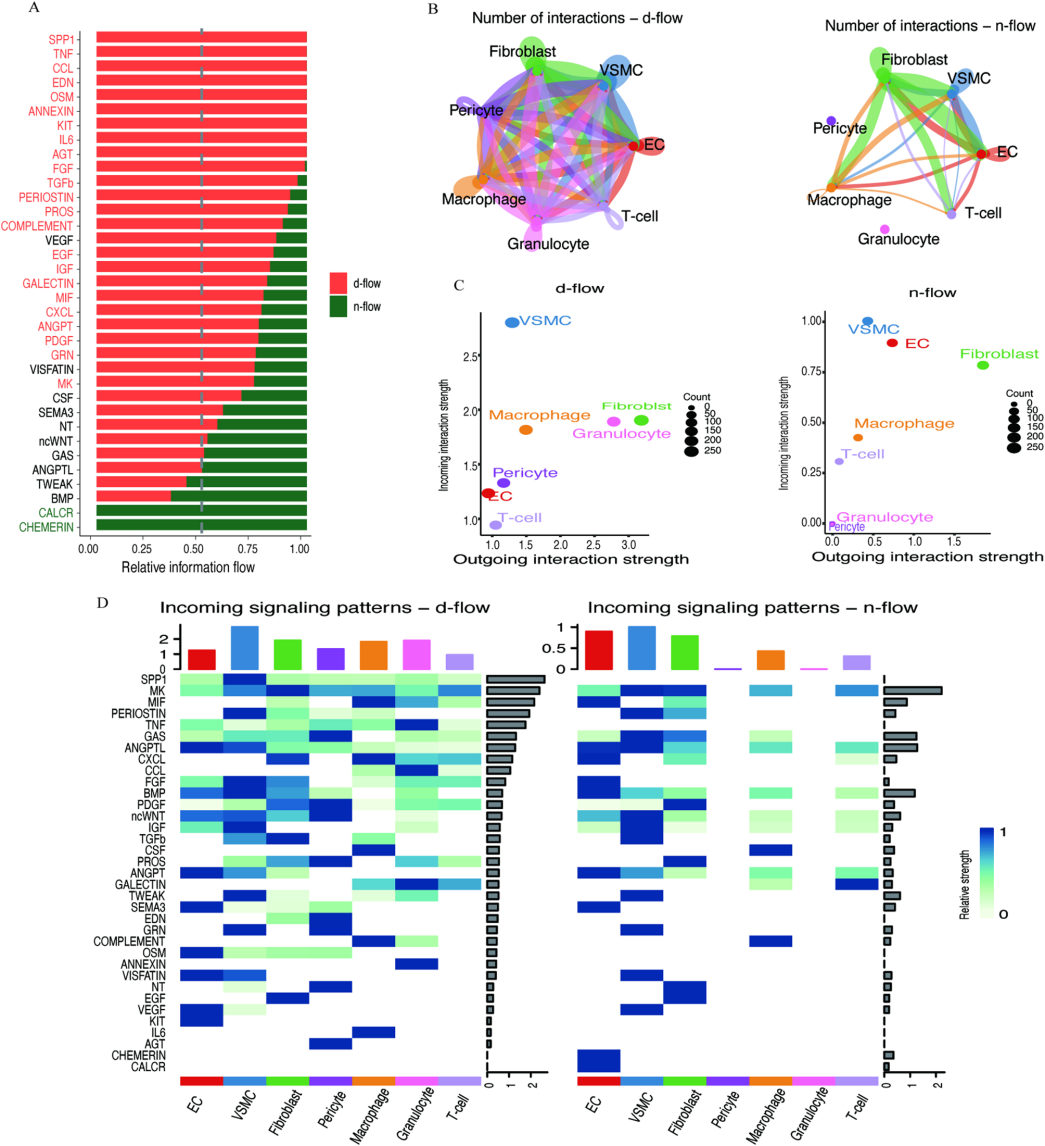
Analysis of cell-cell communication in major cell populations (**A**) Signaling pathways are ranked by differences in overall information flow within inferred networks between d-flow and n-flow groups. Red indicates pathways predominantly enriched in d-flow, black represents pathways equally enriched in both groups, and green signifies pathways enriched in n-flow. (**B**) Circular plot displays the number of interactions between major cell types. Line thickness corresponds to the count of unique ligand-receptor interactions, with loops indicating autocrine circuits. (**C**) Scatter plot illustrates the sources of incoming and outgoing signals for major cell types in both d-flow and n-flow groups. (**D**) Comparison of incoming signaling patterns in cells between the d-flow and n-flow groups, with color intensity indicating the contribution score computed from pattern recognition analysis. A higher score indicates a greater enrichment of the signaling pathway in the respective cell group.

The interaction between cells plays a crucial role in the occurrence and development of atherosclerosis. Cellchat was used for cell communication analysis to understand the changes in cell communication following disturbed flow stimulation. Compared with the n-flow group, the d-flow group showed significant increases in many signaling pathways, including inflammation-related pathways such as TNF, IL6, CCL, and CXCL, as well as TGF-β-related pathways, and signaling pathways regulating endothelial function like EDN, VEGF, TFGB, and OSM pathways (Fig. 8A). In the d-flow group, cell-cell communication was dramatically enhanced, particularly between infiltrating immune cells and vascular wall stromal cells. (Fig. 8B). In addition, it was observed that after disturbed flow stimulation, granulocytes even became one of the primary sources of signal output, second only to fibroblasts (Fig. 8C). Therefore, further investigation into these two cell groups was warranted. Under disturbed flow stimulation, endothelial cells received more VEGF signals (Fig. 8D), which was closely related to endothelial cell proliferation, migration, and endothelial dysfunction. A significant enhancement in the TGF-β signals received by fibroblasts was observed (Fig. 8D). These results further confirmed the previous analysis, suggesting that under disturbed flow stimulation, TGF-β1 originating from macrophages may be involved in the secretion phenotype transition of fibroblasts and contribute to the process of atherosclerosis by activating the TGF-β signaling pathway.

## Discussion

Atherosclerosis tends to occur in regions of disturbed blood flow^9,46^. This study explored how disturbed flow initiate and aggravates atherosclerosis. scRNA-seq identified seven cell clusters in mouse arteries: endothelial cells, VSMCs, fibroblasts, pericytes, macrophages, neutrophils, and T cells. Endothelial cells, VSMCs, and fibroblasts had the highest enrichment of CVD risk gene scores. A distinct fibroblast subgroup (C_3) displayed high enrichment in inflammation and ossification-related pathways. CD36^+^ endothelial cells exhibited significant senescence phenotypes following disturbed flow stimulation. VEGFA^+^ macrophages were increased in the disturbed flow stimulation group, displaying pronounced M1 pro-inflammatory phenotype associated with the severity of atherosclerosis and plaque rupture.

The senescence of endothelial cells play a crucial role in atherosclerosis development^54,55^. Due to telomere shortening, senescent endothelial cells undergo a series of changes that can affect the function of the endothelial nitric oxide (NO) synthase (eNOS), leading to a reduction in NO release, causing abnormal vascular relaxation and constriction^7^. Furthermore, senescent endothelial cells can have elevated expression of adhesion molecules, like ICAM-1 and VCAM1, and disrupted tight junctions, thereby causing impairment to the endothelial barrier function^6,56^. In turn, it increases the infiltration of immune-inflammatory cells such as monocytes and lymphocytes, as well as the deposition of LDL, setting off a cascade reaction leading to atherosclerosis plaque^57^. However, research on the impact of disturbed flow stimulation on endothelial cell senescence in vivo is currently lacking. This study obtained scRNA-seq data of disturbed flow stimulation from public database to explore the phenotypic changes of carotid arteries under disturbed flow stimulation. Endothelial cells and VSMCs exhibited signs of senescence after disturbed flow stimulation, with the changes in endothelial cells being the most significant. Furthermore, disturbed flow stimulation induced the formation of CD36^+^ endothelial cells, which corresponds to the previously observed enrichment of CD36^+^ endothelial cells in the physiologically disturbed regions of the aortic arch’s inner curvature. These results suggest targeting these specific endothelial cells could potentially slow down the progression of atherosclerosis.

Using scFEA analysis, this study unveiled the metabolic changes in the major cell populations of the carotid artery following disturbed flow stimulation. Consistent with a previous study^58^, endothelial cells exhibited enhanced glycolytic pathway activity and decreased oxidative phosphorylation after disturbed flow stimulation. Nevertheless, the most dramatic metabolic changes occurred in fibroblasts. Li et al.^59^ documented that in pulmonary hypertension, adventitial fibroblasts experience metabolic reprogramming, leading to a heightened dependence on aerobic glycolysis over oxidative phosphorylation. This shift in metabolic preference is believed to facilitate the necessary anabolic processes for proliferation and inflammation. Similarly, these metabolic pathway changes may serve as the underlying reasons for the phenotypic changes in fibroblasts under disturbed flow stimulation and play a crucial role in atherosclerosis progression.

Previous research on GWAS data has yielded a plethora of single-nucleotide polymorphisms (SNPs) associated with CVDs^60–62^. Using methods based on expression quantitative trait loci (eQTL)^63^ could aid in identifying gene expression changes co-located with SNPs in specific tissues. In addition, transcriptome-wide association studies (TWAS)^64^ could assist in identifying gene expression changes associated with disease phenotypes at the tissue level. Nevertheless, both approaches overlook gene expression heterogeneity among different cell types within the tissue and may easily miss the contributions of less prevalent but crucial cell subpopulations. This study employed single-cell methods to delve into the differences in gene expression among various cell populations in CVD risk genes and examined the changes in expression following CVD risk factors such as disturbed flow stimulation.

In previous single-cell studies on atherosclerosis, various macrophage subtypes have been identified, including TREM2hi Mφ (high expression of Abcg1, Trem2, Fabp4, Cd9, Spp1, Mmp12, and Mmp14), SMC-derived Mφ (Klf4, Cd68, Lgal3), Proliferative Mφ (Mki67, Birc5, and Stmn1), inflammatory Mφ (Cxcl2, Ccl3, Ccl4, Il1β, and Tnf), Adventitia resident (Lyve1, Mrc1, Cx3cr1, Pf4, and Folr2), and IFN-inducible (Irf7, Ifitm3, Isg15, and Ifit2)^27,65,66^. TREM2hi Mφ, for example, has been associated with lipid metabolism capabilities and presents lower inflammation levels^66^. SMC-derived Mφ tends to differentiate into foam cells after extensive lipid ingestion^67^, while adventitia resident macrophages play a crucial role in maintaining vascular homeostasis^68^. However, these studies have primarily focused on macrophages within atherosclerotic plaques, which represent the middle-to-late stages of atherosclerosis. In this study, early-stage infiltrating VEGFA + macrophages characterized by strong phagocytic activity and limited lipid processing capabilities were identified. These macrophages exhibited a high expression of inflammatory factors, resembling M1-like macrophages, particularly the inflammatory Mφ subtype mentioned earlier. Their robust phagocytic activity and limited lipid efflux capacity suggest their potential involvement in necrotic core formation following extensive lipid ingestion in the early stages^27,65,66^. Moreover, these macrophages present pro-angiogenic function, indicating that the process of vascularization occurs in the early stages of atherosclerosis, not solely in the middle-to-late stages after plaque formation. Angiogenesis, the process of new blood vessel formation, has emerged as a critical factor influencing the stability of atherosclerotic carotid plaques. A growing body of evidence suggests that intraplaque neovascularization is strongly associated with plaque vulnerability and the subsequent risk of ischemic events, particularly stroke^69,70^. These neovessels from angiogenesis tend to be larger, more irregular, and located within the plaque and fibrous cap, contributing to plaque instability^71^. Also, their presence contributes to a heightened risk of rupture and thromboembolic complications^72^. A particularly noteworthy aspect of plaque instability is its relationship with intraplaque hemorrhage^73^. These microvessels, often immature and fragile, are prone to leakage or rupture, further exacerbating plaque instability and increasing the likelihood of symptomatic carotid occlusive disease. Mechanistically, plaque angiogenesis is driven by localized hypoxia and inflammatory signaling, which together stimulate the upregulation of vascular endothelial growth factor-A (VEGF-A)^74,75^. VEGF-A, in turn, promotes the formation of immature and leaky microvasculature. In this study, we found VEGFA + macrophage may be an important contributor of VEGFA in atherosclerosis plaques.

Under disturbed flow stimulation, fibroblasts undergo the most significant changes, a cell type that has been overlooked for long time. Our study revealed that fibroblasts undergo a transition towards an osteogenic-like phenotype after disturbed flow stimulation, and this transition could significantly alter the composition of the extracellular matrix in the outer layer. The alteration of the extracellular matrix could further lead to increased stiffness of the arterial adventitia and decreased elasticity^76,77^. However, in our integrated analysis of single-cell samples from human carotid plaques, we did not identify this osteoblast-like fibroblast subtype. A possible explanation is that fibroblasts are typically distributed in the adventitia of arteries, and the human carotid plaque samples usually lack the adventitial layer. Notably, the mouse carotid artery samples analyzed here represent the very early stages of atherosclerosis development, which exhibit fundamentally distinct pathological features compared to advanced human carotid plaques. This temporal disparity suggests that osteoblast-like fibroblasts might represent a transitional cell state during early atherogenesis, whose functional significance diminishes in late-stage disease progression. Also, the cellular origin of these osteoblast-like fibroblasts remains unclear. One possibility is that disturbed flow stimulation induces metabolic reprogramming in quiescent fibroblasts, driving their activation into this functional phenotype^78^. Alternatively, trans-differentiation from endothelial cells or smooth muscle cells should be considered^79–82^. Our data analysis suggested that under disturbed flow stimulation, smooth muscle cells exhibit downregulation of contractile genes (ACTA2, MYH11, TAGLN) alongside upregulation of fibroblast-associated genes (LUM, DCN, FN1). To address these hypotheses, it would be necessary to employ lineage tracing methodologies combined with single-cell sequencing in future studies. Such approaches could definitively resolve whether these osteoblast-like fibroblasts originate from resident fibroblasts, transdifferentiated SMCs/ECs, or represent a hybrid transitional state. It reminds of the importance of the “outside-in” hypothesis in atherosclerosis^83^. In the initial stage of atherosclerosis, adventitia may first sense external stimuli, and adventitial fibroblasts become activated, altering the extracellular matrix composition and secreting chemotactic and inflammatory factors, driving more immune-inflammatory cells from the capillaries within the adventitia to infiltrate the vessel wall^84,85^. In future research, attention should be paid to the role of adventitia fibroblasts in atherosclerosis.

While this integrative single-cell analysis provides mechanistic insights into disturbed flow-induced atherosclerosis, several limitations warrant consideration. Firstly, reliance on public datasets introduces constraints: the mouse carotid ligation model captures early atherogenic events, whereas human endarterectomy specimens represent advanced plaques lacking adventitial fibroblasts, limiting direct cross-species and disease-stage comparisons. Secondly, while computational approaches (scFEA, CytoSig) revealed fibroblast metabolic reprogramming and macrophage-endothelial crosstalk, these predictions require experimental validation through isotopic flux analysis and spatial co-culture systems under controlled shear stress. Lastly, the absence of longitudinal data precludes delineating temporal relationships between endothelial senescence, fibroblast osteogenic transition, and VEGFA + macrophage expansion. Addressing these gaps through multi-omics validation and preclinical models could strengthen the therapeutic relevance of our findings.

## Conclusion

This study systematically elucidated the functional changes of the main cell populations under disturbed flow using scRNA-seq data. We found CD36^+^ endothelial cells, VEGFA^+^ macrophages and adventitial fibroblasts play critical roles in the occurrence and progression of atherosclerosis. These findings may provide inspiration for research on atherosclerosis pathogenesis and targeted treatments.

## Electronic supplementary material

Below is the link to the electronic supplementary material.

Supplementary Material 1

## Data Availability

The datasets analyzed during the current study are available in the SRA and GEO repositories, including PRJNA722117, GSE159677, GSE43292, GSE163154, and GSE41571.

## Abbreviations

AC: Atherosclerotic core
CVD: Cardiovascular disease
d-flow: Disturbed flow
EndMT: Endothelial to mesenchymal transition
eQTL: Expression quantitative trait loci
GO: Gene Ontology
GSEA: Gene set enrichment analysis
GWAS: Genome-wide association study
HGVs: Highly variable genes
LDL: Low-density lipoprotein
MSigDB: Molecular Signatures Database
n-flow: Normal flow
PA: Adjacent portion
scRNA-seq: Single-cell RNA sequencing
TF: Transcription factor
TWAS: Transcriptome-wide association studies
UMAP: Uniform manifold approximation and projection
