# Supplementary material for: Single-cell RNA-seq analysis of mouse carotid artery under disturbed flow and human carotid plaques identifies key cell populations in atherosclerosis development
Source: Sci Rep. 2025 Jul 1;15:20747. doi: 10.1038/s41598-025-07395-7 (PMC12214537; doi:10.1038/s41598-025-07395-7)
Supplement: Supplementary file 1 — Supplementary Material 1 [file 41598_2025_7395_MOESM1_ESM.pdf]

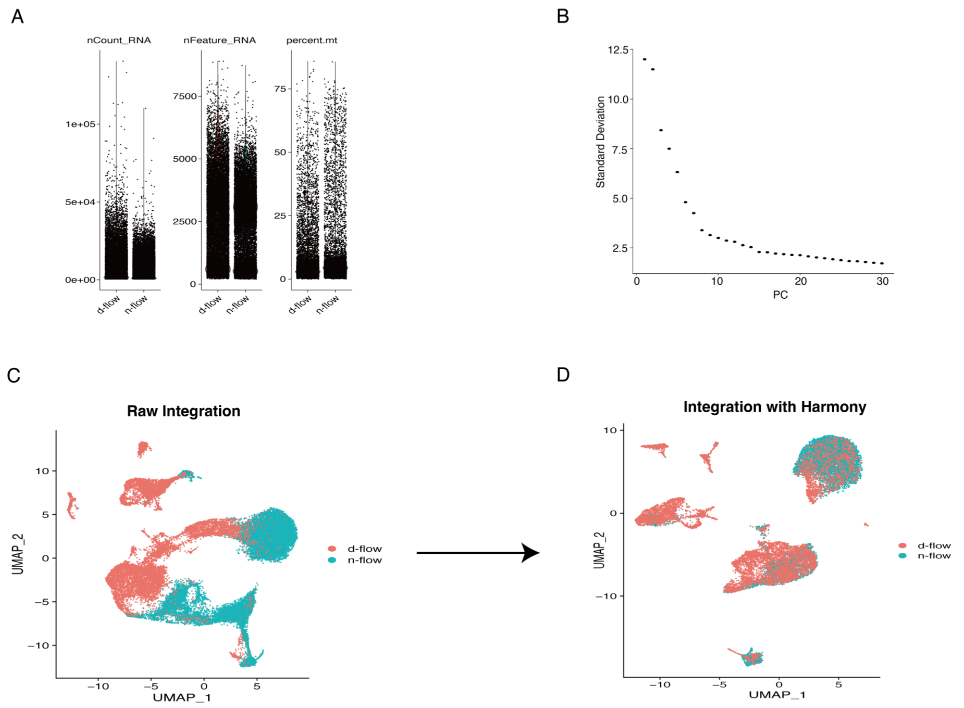


**Supplementary Figure S1. Quality control of mouse PCL data. A.** Vlnplot of nCount_RNA, nFeature_RNA and percent.mt between d-flow and n-flow group. **B.** Elbowplot show standard deviation of first 30 PCs. **C.** Umap plot of combined datasets (d-flow group and n-flow group) colorized by data source. **D.** Umap plot of combined datasets (d-flow group and n-flow group) remove batch effect by Harmony.

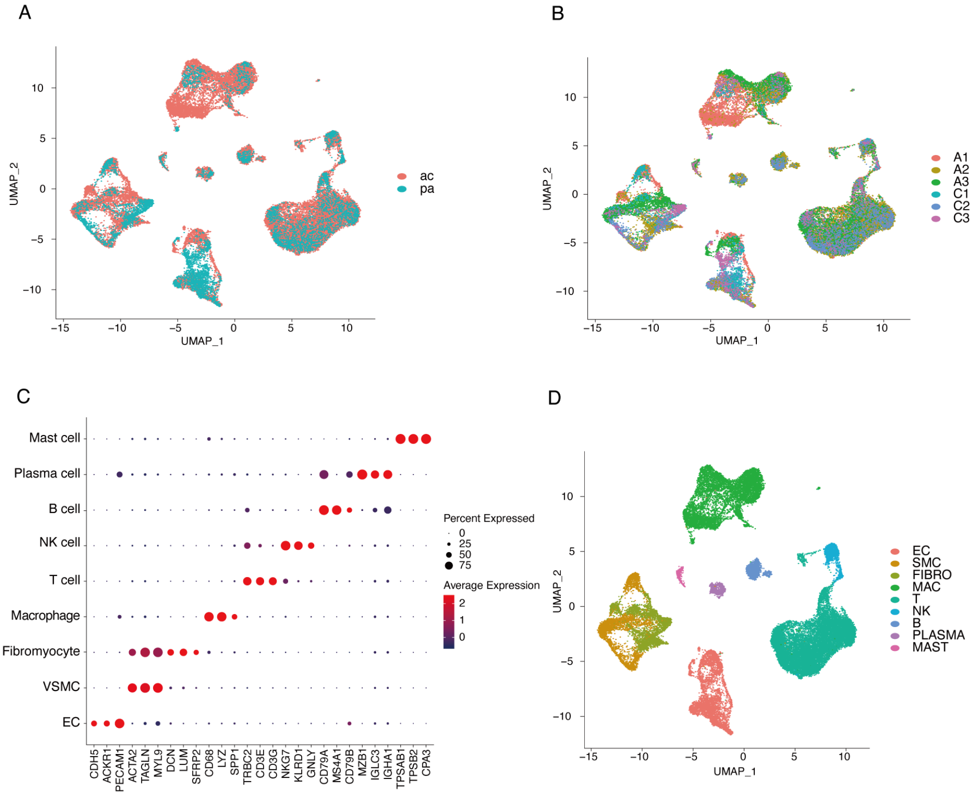


**Supplementary Figure S2. Quality control and celltype annotation of human carotid plaque single cell RNAseq data. A.** Umap plot of combined datasets colorized by plaques distribution. B. Umap plot of combined datasets colorized by single patient. C. Dotplot showing average expression of known markers in main cell types. D. Umap plot show the landscape of main cell types.

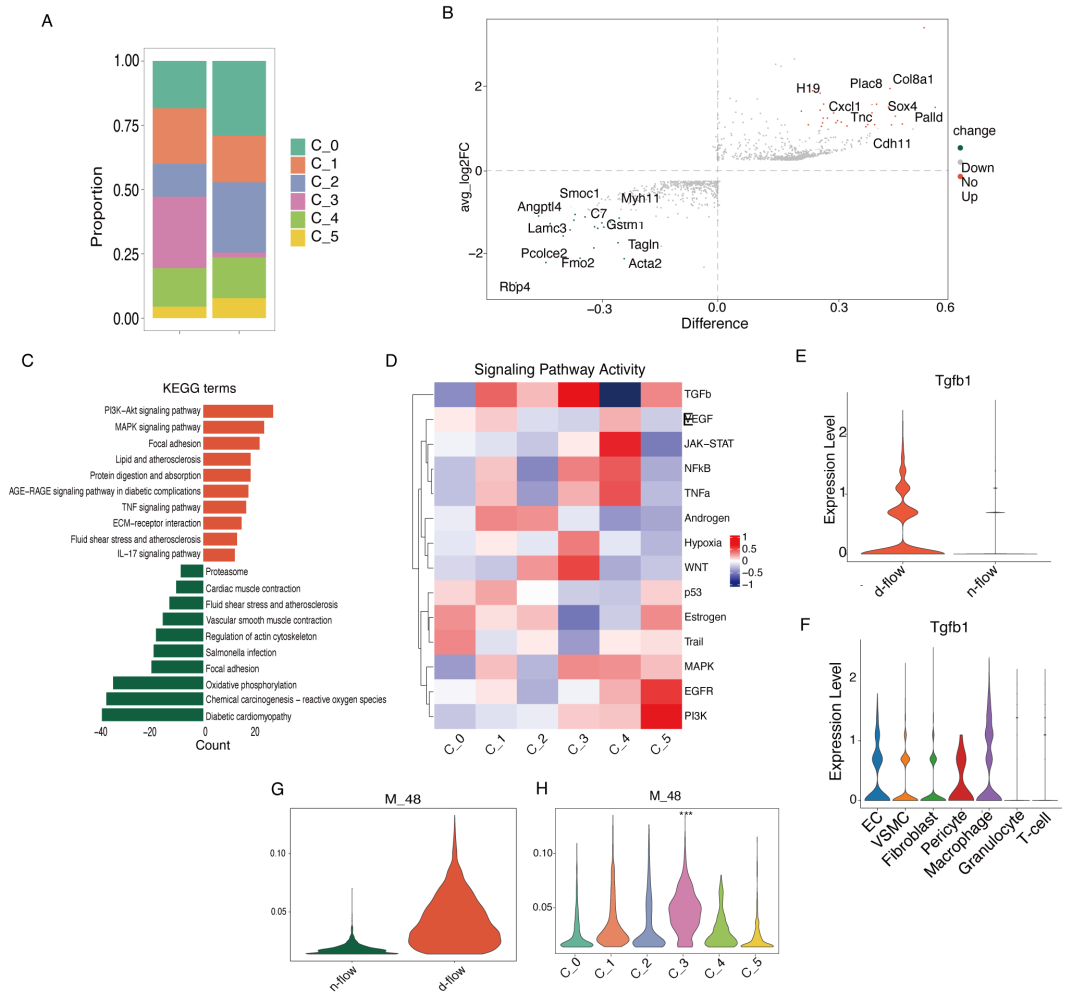


**Supplementary Figure S3. A.** Stacked bar plot showing the proportion of subclusters in two groups. **B.** Volcano plot highlighting disparities in gene expression within fibroblasts, with a focus on genes exhibiting differential expression (*P*_val_adj < 0.05). **C.** KEGG enrichment analysis of both upregulated and down-regulated genes. **D.** Heatmap representing the activity of signaling pathways within fibroblast subclusters, as computed using the “progeny” method. **E. F.** Violin plot concentrating on the expression of the Tgfb1 gene. **G. H.** Vlnplot show the activity of M_48.

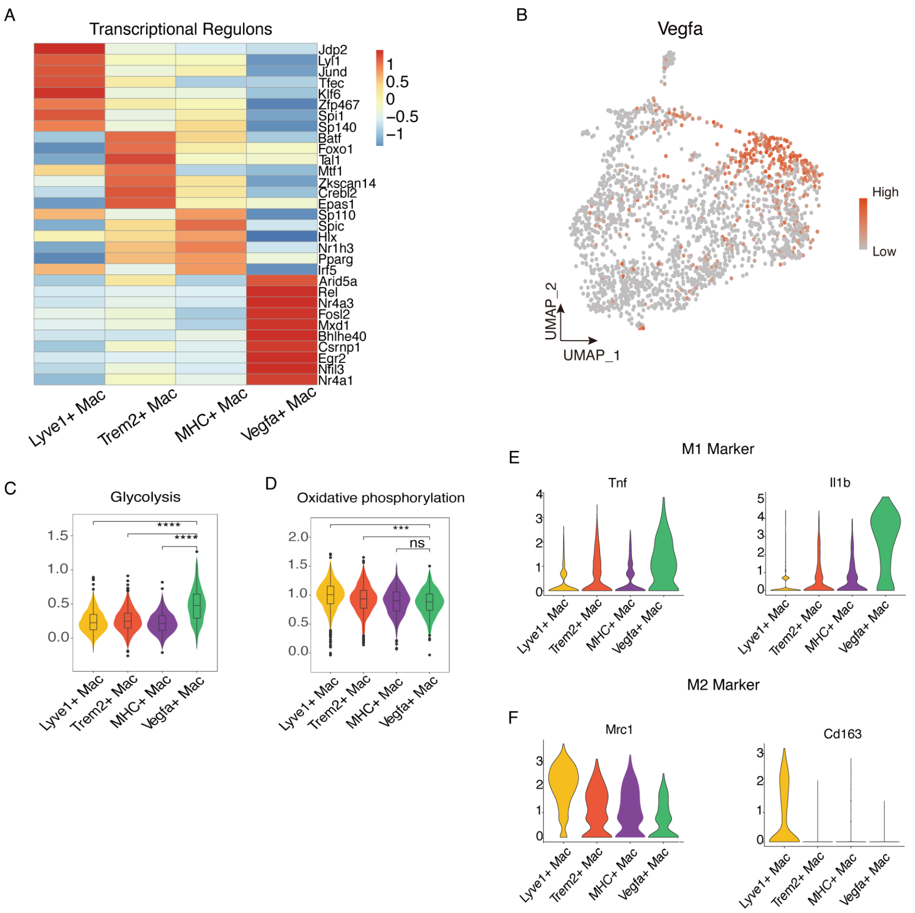


**Supplementary Figure S4. A.** Heatmap displaying the main transcription factor regulons across four macrophage clusters. **B.** Featureplot visualizing the expression of the vascular endothelial growth factor a (VEGFA) gene. **C** and **D.** Vlnplots presenting glycolysis score (left) and oxidative phosphorylation score (right). **E** and **F.** Vlnplots representing M1-like macrophage score and M2-like macrophage score.

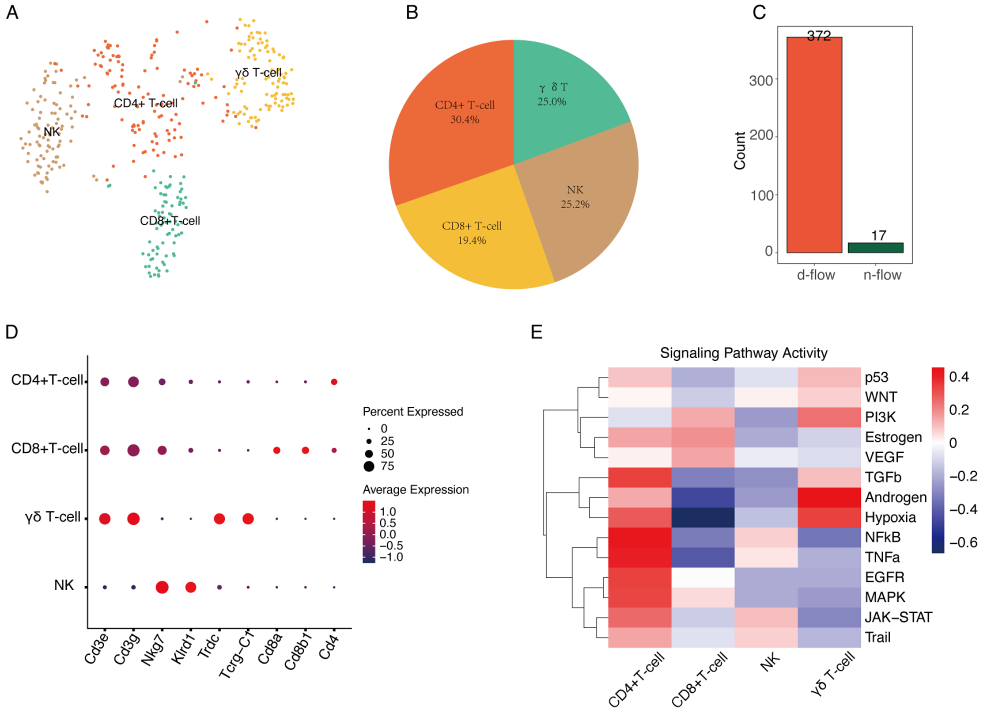


**Supplementary Figure S5. Identifying T cell infiltration in the carotid artery following disturbed flow stimulation. A.** Umap plot to visualize the distribution of cell types. **B.** Pie chart representing cell type proportions in the d-flow group. **C.** Bar Plot comparison of the total T cell counts across different groups. **D.** Dot Plot to visualize marker gene expression patterns. **E.** Progeny analysis to quantify signaling pathway activity using the Progeny package.
